# Hypotension and Bradycardia Produced by Transthoracic Application of Low-Intensity Ultrasound Therapy in Hearts of Healthy Rats - A Preclinical Study

**DOI:** 10.21470/1678-9741-2019-0255

**Published:** 2020

**Authors:** Washington Luiz Silva Gonçalves, Anabel Nunes Rodrigues, Rodrigo Chaves, Sonia Alves Gouvea

**Affiliations:** 1 Laboratório de Inovações Tecnológicas no Ensino em Saúde - LITES, Universidade Santa Úrsula - USU, Botafogo, RJ, Brasil.; 2 Departamento de Ciências Fisiológicas, Programa de Pós-Graduação em Ciências Fisiológicas, Universidade Federal do Espírito Santo - UFES, Vitória, ES, Brasil.

**Keywords:** Ultrasonic Waves, Hemodynamics, Femoral Artery, Capillary Permeability, Bradycardia, Hemodynamics/ Catheterization

## Abstract

**Objective:**

To investigate the cardiovascular effects produced by transthoracic application of low-intensity pulsed ultrasound therapy (LIPUST).

**Methods:**

Three-month-old male Wistar rats (± 300 g, N=16) were randomly allocated in two groups, namely SHAM (control group, faked procedures) and UST (animals treated with LIPUST). These animals, under anesthesia, were instrumented (femoral artery and vein catheterization) for hemodynamic recordings (mean blood pressure [MBP], heart rate [HR]) and blood biochemical profile (lipids, creatine kinase-myocardial band [CK-MB]). Then, LIPUST (spatial average-temporal average [ISATA] 1-MHz, power 0.1 to 1.2 W/cm2, pulsed 2:8 ms, cycle at 30%, for three minutes) was applied to animals from the UST group, externally to their thorax. SHAM animals were equally manipulated, but without application of ultrasound energy. After the hemodynamic and biochemical measurements, animals were sacrificed, and their hearts were mounted in a Langendorff apparatus for coronary reactivity evaluation. Standard histology techniques were employed to analyze the hearts.

**Results:**

LIPUST application caused statistically significant reductions in MBP (92±4 *vs*. 106±1 mmHg) and HR (345±14 *vs*. 380±17 rpm) when compared with SHAM procedures. UST rats exhibited higher CK-MB levels (318±55 *vs*. 198±26 U/dL) and lower plasma triglycerides levels (38±7 *vs*. 70±10 mg/dL) than SHAM animals. Coronary reactivity was not significantly changed by LIPUST. Cardiac histopathology showed an increase in capillary permeability in treated animals when compared with SHAM animals.

**Conclusion:**

Noninvasive LIPUST induces significant metabolic and hemodynamic changes, including intensity-dependent bradycardia and hypotension, indicating a possible therapeutic effect for cardiac events.

**Table:** 

Abbreviations, acronyms & symbols				
**ANOVA**	**= Analysis of variance**		**LIPUST**	**= Low-intensity pulsed ultrasound therapy**
**CK**	**= Creatine kinase**		**MBP**	**= Mean blood pressure**
**CK-MB**	**= Creatine kinase-myocardial band**		**NO**	**= Nitric oxide**
**CPP**	**= Coronary perfusion pressure**		**PE**	**= Polyethylene**
**CRP**	**= C-reactive protein**		**SHAM group**	**= Control group, faked procedures**
**HDL-C**	**= High-density lipoprotein cholesterol**		**TC**	**= Total cholesterol**
**HE**	**= Hematoxylin-eosin**		**TG**	**= Triglycerides**
**HR**	**= Heart rate**		**US**	**= Ultrasound** *
**ISATA**	**= Spatial average-temporal average intensity**		**UST group**	**= Animals treated with LIPUST**
**LDL-C**	**= Low-density lipoprotein cholesterol**			

## INTRODUCTION

In recent years, the effects of application of ultrasound (US) energy to various biological systems have been widely studied^[1-8,11-25]^. This has been particularly seen in clinical and experimental cardiology, by using various intensities (power, frequency, time) of the US for both diagnostic and therapeutic purposes^[11,14-16,19,22]^.

Experimental and clinical investigations have shown that application of therapeutic US to the thorax irradiates to the heart, lungs, and great vessels, causing several effects, including mild vascular thrombolysis^[22-24]^, fibrinolysis activation^[6,22]^, angiogenesis^[4]^, inotropic and lusitropic effects^[7,22]^, reduction of reperfusion-induced arrhythmias^[5]^, coronary vasodilatation^[14,16,18]^, and release of bioactive substances^[1,2,13,22]^. US energy can also cause drug phonophoresis in humans^[21]^ and it is synergistic with some vasodilator agents^[20]^. These systemic effects of US, which possibly reflect primary actions into individual cells and/or subcellular structures, strongly depend on the parameters of the dose (*i.e.*, power, frequency, and duration) irradiated^[19,25]^. However, the interdependence of these parameters is still a matter of investigation by using both experimental models and human studies.

In fact, very few studies have addressed the relationship between US parameters and their biological consequences, including the molecular and cellular mechanisms involved. This preliminary study was therefore designed to further investigate the cardiovascular effects of noninvasive transthoracic low-intensity pulsed ultrasound therapy (LIPUST) in healthy rats.

## METHODS

### Animals and Groups

All procedures were performed in accordance with the international guidelines, particularly to those established in the Guide Care and Use of Laboratory Animals (1996) and in the Brazilian Guideline for the Care and Use of Animals for Scientific and Educational Purposes - DBCA, of the National Council for the Control of Animal Experimentation - CONCEA (2013), and approved by Institutional Ethic Committee - CEUA (number# 059/2012 and revised 2018).

Three-month-old male Wistar rats weighing 300 to 350 g, from our breeding stock, were divided into two groups, namely UST (animals treated with LIPUST) and SHAM (control group, faked procedures) (N=8 per group). Throughout the experiment, the animals were individually housed in Perspex^®^ cages with sawdust bedding material, in a noiseless room, at 22-24 ºC, with lights on from 6:00 h to 18:00 h. Pelleted food and filtered water were freely available. Animals were maintained anesthetized with halothane-3% (vaporized from a contention chamber) after induction with a mixture of ketamine and xylazine (respectively, 10 and 50 mg.kg-1, intraperitoneally). While anesthetized, it was recorded their body weight and nose-anus length. A trichotomy of 3 cm^2^ in the precordial area was then made and the Protocol 1 was conducted (see below).

### Protocol 1 (*In Vivo*): Hemodynamics and Biochemical Measurements

Under anesthesia, the animals had their right femoral artery and vein cannulated (polyethylene [PE]-10, PE-50 tubes) (Clay Adams, United States of America) for hemodynamic recordings and blood samples, respectively. Each PE catheter was flushed and filled with 40 U/mL heparinized saline. Arterial catheter was connected to a pressure transducer (Statham model P23Db) coupled to a Power-Lab^TM^ (ADI Instruments, Bela Vista, NSW, Australia) to record mean blood pressure (MBP) and heart rate (HR). A calibrated digital infrared thermometer (OS-VIR50, Omega™, São Paulo, Brazil) was also used to measure body temperature (chest skin) during the application of LIPUST.

**Fig. 1:**
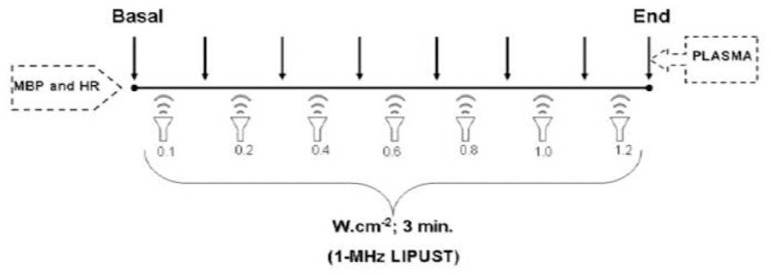
Schematic illustration of the experimental protocol for noninvasive transthoracic low-intensity pulsed ultrasound therapy (LIPUST) to healthy Wistar rats. HR=heart rate; MBP=mean blood pressure; trace=hemodynamic record; arrows=time of the hemodynamic and plasma measures.

LIPUST (or sham stimulation) was then externally applied (noninvasively) to the animal precordial area (3 cm^2^), as follows: (a) sonogel application; (b) transducer (1-MHz) positioned at 0.1 cm from the thoracic skin; and (c) US (or sham) application, using an AVATAR II™ equipment (KLD Biosystems, São Paulo, SP, Brazil). Irradiation parameters were spatial average-temporal average intensity (ISATA) = 1 MHz (kept constant) at increasing powers (0.1, 0.2, 0.4, 0.6, 0.8, 1.0, and 1.2 W.cm^2^) pulsed with 2:8 ms, cycles at 30%, and three-minute duration with five-minute time interval between US intensities. The transducer was checked as recommended^[10]^, and the level of irradiation was chosen according to the international guidelines for therapeutic US utilization, such as those from the World Federation of Ultrasound in Medicine and Biology^[3,25]^. Blood samples were collected from the venous catheter at the end of the *in vivo* experiment to perform biochemical determinations (Figure 1).

Using conventional analytical laboratory methods, the following substances were determined in plasma: (a) total cholesterol (TC); (b) low-density lipoprotein cholesterol; (c) high-density lipoprotein cholesterol; (d) triglycerides (TG); (e) total creatine kinase (CK), (f) CK-myocardial band (CK-MB), and (h) C-reactive protein (CRP). In sequence, Protocol 2 was performed^[26]^.

### Protocol 2 (*In Vitro*): Isolated Perfused Heart

The animals were sacrificed under halothane (100%) anesthesia, had their thorax opened, and their heart was carefully transferred to a Langendorff perfusion system (Hugo Sachs Electronics, March-Hugstetten, Germany).

The isolated hearts were perfused with a modified Krebs solution (Sigma-Aldrich, St. Louis, MO, United States of America), maintained pH 7.4, and kept at 37 ºC. The solution was equilibrated with a carbogen mixture (O_2_ 95% + CO_2_ 5%) (White-Martins, Rio de Janeiro, Brazil). The perfusion flow was maintained at 10 mL/min by a peristaltic pump (4-channel MS-Reglo, Hugo Sachs Electronics).

**Fig. 2:**
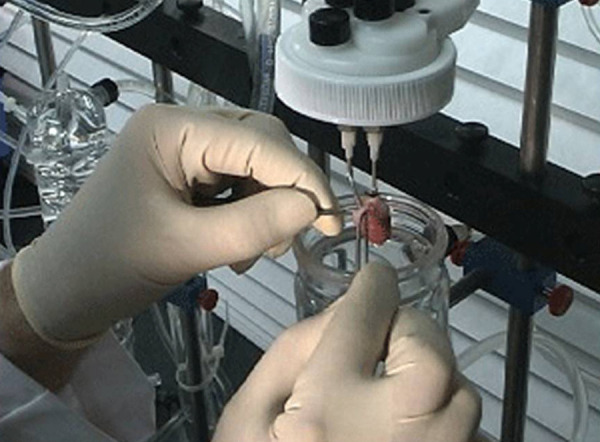
Photograph of the isolated heart study (Langendorff method), experimental protocol 2 "in vitro” or “ex vivo", time of heart connection to data acquisition equipment.

A small latex balloon filled with saline (10 mmHg pressure) was carefully introduced into the left ventricle. A steel catheter connected the latex balloon to a mechanical transducer (TPS-2 Statham transducer, Incor, São Paulo, Brazil), to allow recording the isovolumetric cardiac contractility. The coronary perfusion pressure (CPP) was continuously recorded by another transducer connected to a data acquisition system Power-Lab™ (ADI Instruments, Bela Vista, NSW, Australia). After 40-minute stabilization, basal CPP was determined and a dose-response curve to bolus injections of adenosine 1, 4, 8, 16, 32, 64, 128, and 256 µg (Sigma-Aldrich, St. Louis, MO, United States of America) was obtained to evaluate the coronary reactivity (Figure 2).

### Histopathology

At the end of the perfusion experiment, the hearts were removed and were fixed for 24 h in 10% formalin solution (pH 7). Thereafter, the myocardial samples were gradually dehydrated with increasing concentrations (70 to 100%) of ethanol, clarified in xylene, and embedded in paraffin blocs, to allow microtomy. Sections of 6 µm thickness were made with a microtome (Spence 820, New York, NY, United States of America) and stained with hematoxylin plus eosin to allow histological analysis. Capillary histomorphometry was performed from pictures captured by a 40x objective; evaluation was made using a computerized imaging processing system (Sigma-Pro™ Image; St. Louis, MO, United States of America). Five pictures from samples from pairs of tissue (*i.e.*, SHAM and UST) were randomly captured under optical microscopy (Olympus™ AX70 Plus, Tokyo, Japan) and were stored for further analysis. Such analysis included a semiquantitative determination of myocardial capillarity and interstitial area, by using digital markers (as color contrasts) as discrimination parameters^[9]^.

### Limitations

In order to minimize the use of experimental animals (N=16), according to the institutional ethic committee, the same hearts (from the UST group) were submitted to the US irradiation at seven different powers (21-minute effective time). This fact could have magnified the observed hemodynamic and cellular effects. Another limitation was the removal of a single blood sample from each animal, to minimize the changes in blood volume. If additional samples could have been taken, perhaps a better idea of the time-effects of biochemical changes could be obtained.

### Data Analysis

Data was put into appropriate spreadsheets and analyzed by a Prism-6™ statistical package (GraphPad Software, Inc., San Diego, CA, United States of America). Parametric analyses were Student’s *t*-test (paired and non-paired), analysis of variance (ANOVA) (one- and two-way), and the post hoc Tukey’s test, as appropriate. Nonparametric analysis for semiquantitative data was done by the Mann-Whitney U test. Unless otherwise stated, data are presented as means ± standard error. The level of significance for statistical differences was set at *P*<0.05.

## RESULTS

**Fig. 3:**
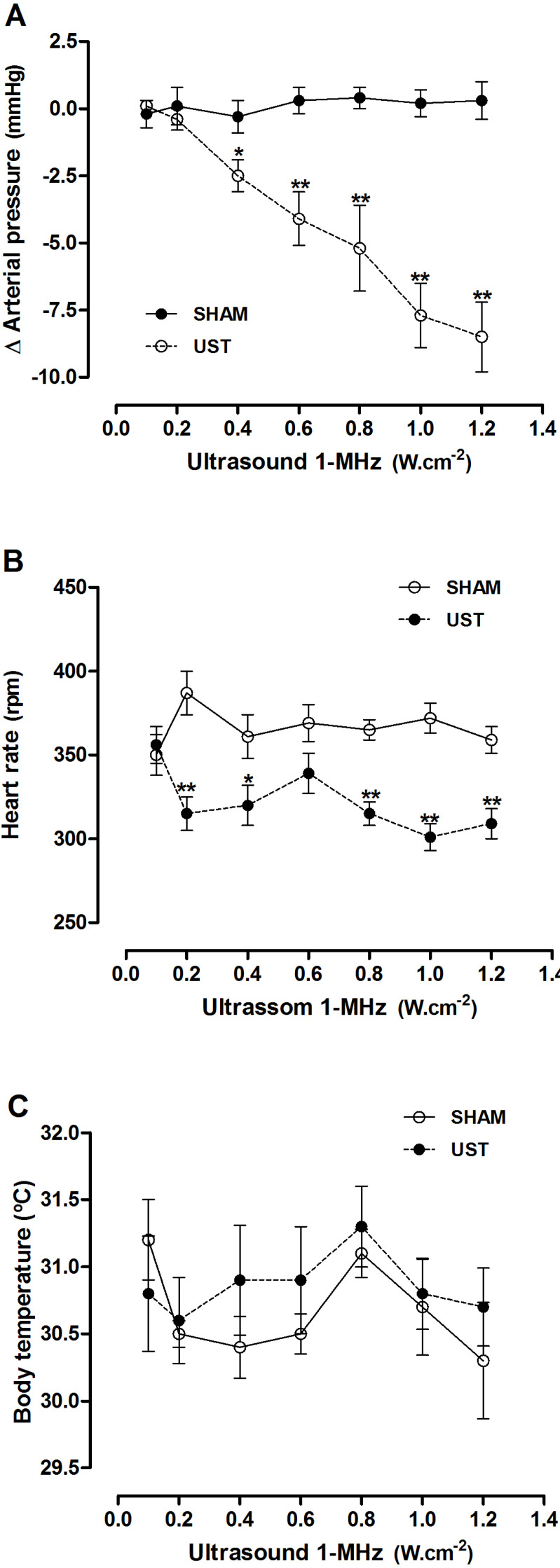
Dose-response curves for (A) mean blood pressure, (B) heart rate, and (C) body temperature changes induced by noninvasive transthoracic low-intensity pulsed ultrasound therapy (LIPUST) (at increased powers) in healthy Wistar rats. Data presented as mean ± standard error. *P<0.05 and **P<0.01, analysis of variance and Tukey’s test as post hoc (N=8 per group). SHAM=control group, faked procedures; UST=animals treated with LIPUST.

In the *in vivo* studies, the dose-response curve for application of LIPUST to the heart (UST group) showed a progressive reduction (*P*<0.05 and *P*<0.01) in MBP (ΔMBP) from the third US dose (Figure 3A). The HR curve also showed a reduction (*P*<0.01) in this parameter in the UST group, which was already observed from the first US dose (Figure 3B). The body temperature curve showed no significant changes, there was a mean variation of ± 0.6 ºC in the animals from SHAM and UST groups (Figure 3C).

**Fig. 4:**
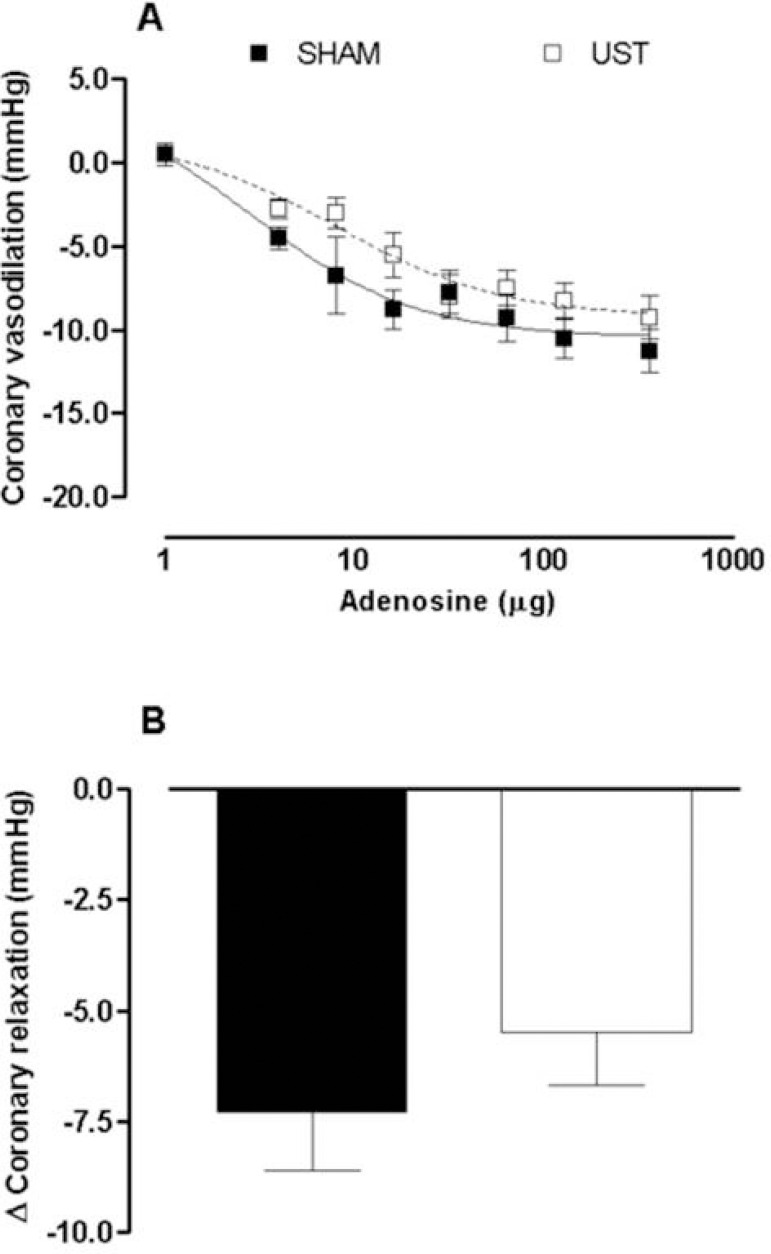
Coronary vasodilatation produced by adenosine (A) and changes in basal coronary pressure (B), after noninvasive transthoracic low-intensity pulsed ultrasound therapy (LIPUST) (at increased powers) in healthy Wistar rats. (N=8 per group). SHAM=control group, faked procedures; UST=animals treated with LIPUST.

In comparison, similar curves from SHAM animals did not show any obvious change in the hemodynamic parameters (ΔMBP, HR). Table 1 shows that values of TC and fractions in the UST group were similar to those from the SHAM group. It was also observed that despite the apparent increase in CRP values, there were no significant differences between SHAM and UST animals. However, it was verified that TG decreased (*P*<0.05) in the UST group and that CK and CK-MB enzymes significantly increased (*P*<0.01). On the other hand, the *in vitro* studies on the coronary reactivity did not show any statistically significant difference, regarding the coronary vasodilator effect of adenosine, between SHAM and UST groups (Figure 4A). In addition, the effects of noninvasive LIPUST on the changes in CPP (ΔCPP) were not statistically different of those from SHAM animals (Figure 4B).

**Table 1:** Plasma enzymatic and biochemical profile of SHAM and UST rats.

Plasma parameters	SHAM group	UST group
TC (mg/dL)	53±2	52±2
TG (mg/dL)	70±10	38±7*
HDL-C (mg/dL)	23±1	22±1
LDL-C (mg/dL)	108±9	106±11
Total CK (U/dL)	208±9	510±12**
CK-MB (U/dL)	198±26	318±55**
CRP (mg/dL)	0.31±0.16	0.63±0.22

Data presented as means ± standard error. CK=creatine kinase; CK-MB=creatine kinase-myocardial band; CRP=C-reactive protein; HDL-C=high-density lipoprotein cholesterol; LDL-C=low-density lipoprotein cholesterol; SHAM=control group, faked procedures; TC=total cholesterol; TG=triglycerides; UST=animals treated with low-intensity pulsed ultrasound therapy

* *P*<0.05 and

** *P*<0.01, Student's *t*-test (N=8 per group).

**Fig. 5:**
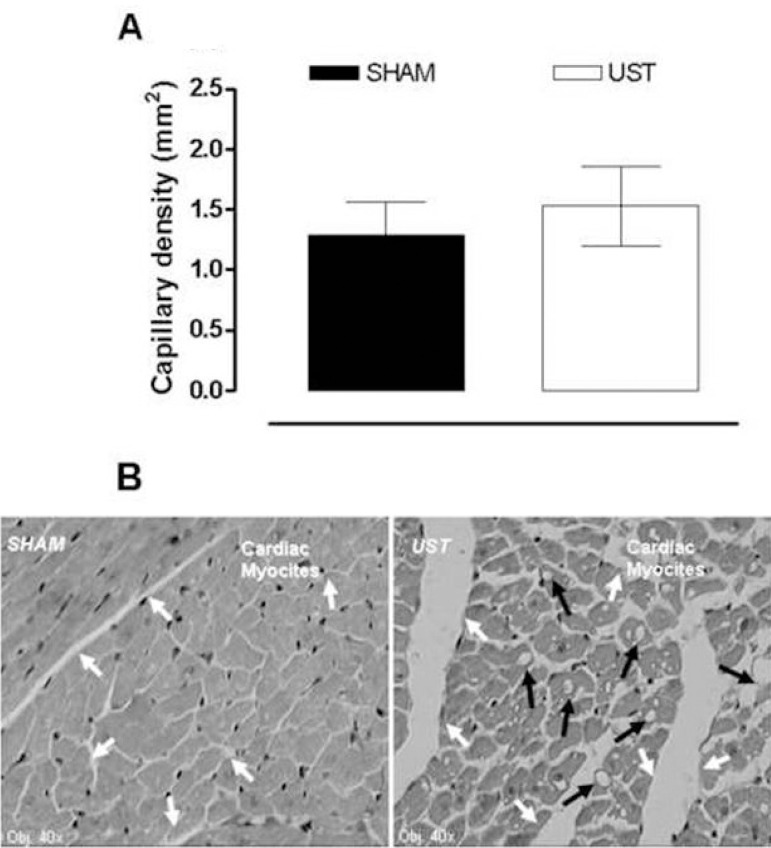
Morphological measures of the myocardial capillary density (A) and myocardial photomicrograph (B) obtained from control (SHAM) and treated with noninvasive low-intensity pulsed ultrasound therapy (UST) animals. (HE; original magnification-40´). White arrows denote increase in interstitial capillary permeability; black arrows denote intracellular lipid deposits. (N=8 per group).

The morphometric analysis of the hearts from the two groups regarding the capillary density also did not show any obvious difference between SHAM and UST groups (Figure 5A). Interestingly, however, the hearts treated with LIPUST displayed a greater capillary permeability than SHAM animals and showed small, yet consistent, lipid deposits inside the myocardial cells (Figure 5B).

## DISCUSSION

In the original and present *in vivo* and *in vitro* studies, we show some cardiovascular effects elicited by transthoracic LIPUST to the heart (via extracorporeal application) from healthy Wistar rats. In our opinion, the main finding of this study was that LIPUST is able to cause parallel reductions in MBP and HR, without important changes in the coronary function. According to Kuma et al.^[14]^, application of low-intensity US energy to open thorax of guinea pigs has produced positive inotropic and lusitropic effects. Such findings are similar to our results in rats, in particular with respect to the power-dependency of the systemic hypotension and bradycardia; they also corroborate other studies using distinct parameters of US energy^[4,5,14,16,19,22]^.

Most of the studies using changes in frequency or time (doses) of US energy, which were interested in verify cardiac effects, were not able to detect systemic hemodynamic implications, as described here; also, most of these studies were done with invasive techniques or using *in vitro* and *in vivo* methods. Such invasive procedures may be associated with post-surgery intercurrences as well as a greater probability of infections^[14,22-24,26]^. In this aspect, the noninvasive LIPUST technique here presented is capable of minimize such adverse effects. However, technical problems persist, since the larger intensities variability and the possibility of activation of multiple biochemical and biomechanical pathways make it difficult to interpret the data, including differing the direct from the indirect effects of the radiation^[12]^. It deserves mention, however, that some of these mechanisms were already described in the literature, including direct vibration effects (mechanical) on the cellular structure and changes in the turn-over of bioactive substances (biochemical)^[7,20,25]^.

The observed reduction in HR (Figure 3) found in the present study can be attributed to the direct mechanical activation of a cardiovascular reflex (baroreflex and Bezold-Jarisch reflex), with a subsequent increase in its sensitivity, although the pathways involved cannot be identified in this study. This hypothesis of involvement of cardiovascular reflexes, especially baroreflex and Bezold-Jarisch reflex, was partially supported by the experiments performed by Miwa et al.^[17]^, who described an increase in sympathetic nerve activity, accompanied by increased norepinephrine release, following application of US energy to subcutaneous adipose tissue in rats. However, one possibility would be that direct stimulation (cumulative tension) of the aortic baroceptors by transthoracic US could trigger the baroceptor reflex, which results in increased parasympathetic tone and reduced sympathetic tone, with consequent bradycardia^[17,22,27-29]^. Another possibility would be the Bezold-Jarisch reflex, a cardiac inhibitory reflex that results in bradycardia, vasodilation, and hypotension, and originates in cardiac receptors, located mainly in the left ventricular posteroinferior wall, which are activated by mechanical-chemical inputs, leading to increased parasympathetic activity and inhibition of sympathetic activity^[17,22,24,26-29]^.In this study, it was not intended to determine the direct mechanisms responsible for the negative chronotropic effects of US on the heart "*in vivo*". However, the observation that these chronotropic effects were mainly restricted to the period of application is suggestive of direct chemical-mechanical action of ultrasonic waves in the heart tissue.

In this context, the concomitant participation of the sonomechanical and sonochemical effects (*e.g.*, flow and microflow) of the ultrasonic therapy is well described and evidenced in this study. In addition, they corroborate the observed changes in plasma concentrations of important biochemical markers such as TG, CK, and CK-MB (Table 1), as the literature has shown that therapeutic US is capable of modifying various enzymatic^[2,11,13,26]^ activities and causing release of intracellular molecules, depending on the US energy^[6,23,24,26,31]^. It is worth mentioning that therapeutic US is capable of activating the release of nitric oxide (NO) and possibly other vasoactive endothelial substances, as well as activating enzymes (metalloproteinases), which could also at least partially explain the hypotensive effects classified and described in this study^[(1.11.14-16.19,22.24)]^.

It can also be speculated that both negative chronotropic and systemic hypotensive effects occurred by the piezoelectric action of low intensity US energy in cardiac tissue. The literature states that US waves deform muscle structural proteins, mainly collagen and tropomyosin, producing electrical charges (tension), in this case, in the cardiomyocyte, and consequently providing changes in HR, rhythm, and regularity^[11-14,22,24,25,27-29,31,32]^.

It has also been shown that US waves, when focused on peripheral vessels (as a target-tissue), caused activation of tissular plasminogen, which is an important thrombolytic agent^[6,15]^. We can speculate that the effect of plasminogen contributed to reduce the adherence of lipids to the arterial wall and reduced possible injuries to the coronary endothelium. Another relevant finding of the present study was the augmented capillary permeability observed in the photomicrography of treated hearts (Figure 5B), further suggesting that the pulsed US therapy acted as a modulator of some physicochemical process involved in cell permeability and perhaps vasomotricity. Similar studies were conducted by Siegel et al.^[22]^ and other authors^[1,11,14,15,24,27-29]^, showing that the release of NO had an important participation in the capillary permeability effects of US therapy. All together, these data suggest that LIPUST can indeed have good therapeutic potentialities.

Additionally, our histopathological results did not shown significant changes in capillarity with LIPUST; however, we were able to identify small intracellular depositions of lipids, that could possibly be due to reesterification of free fatty acids by the myocardial cells (Figure 5)^[21-25,28,31]^. This speculation is based on the observation that LIPUST reduced the plasma TG together with an increase in the tissue perfusion and in the cell permeability, situations that theoretically would facilitate the intracellular fat deposition, here found in the morphologic study of the hearts.

## CONCLUSION

In summary, the data showed that noninvasive LIPUST, applied to the precordial area of healthy rats, causes power-dependent reductions in both HR an MBP, accompanied by improved myocardial perfusion. Despite its limitations, this study pointed to a good therapeutic potential of noninvasive LIPUST in the primary or secondary treatment of cardiac diseases. To clarify and expand these issues, further studies, especially done in humans and using more sophisticated molecular techniques, will be necessary.

**Table:** 

Authors' roles & responsibilities	
WLSG	Substantial contributions to the conception or design of the work; or the acquisition, analysis, or interpretation of data for the work; Drafting the work or revising it critically for important intellectual content; final approval of the version to be published.
ANR	Drafting the work or revising it critically for important intellectual content; final approval of the version to be published.
RC	Final approval of the version to be published.
SAG	Revising it critically for important intellectual content; final approval of the version to be published.

## References

[1] Altland OD, Dalecki D, Suchkova VN, Francis CW (2004). Low-intensity ultrasound increases endothelial cell nitric oxide synthase activity and nitric oxide synthesis. J Thromb Haemost.

[2] Bandow K, Nishikawa Y, Ohnishi T, Kakimoto K, Soejima K, Iwabuchi S (2007). Low-intensity pulsed ultrasound (LIPUS) induces RANKL, MCP-1, and MIP-1beta expression in osteoblasts through the angiotensin II type receptor. J Cell Physiol.

[3] Barnett SB, ter Haar GR, Ziskin MC, Rott HD, Duck FA, Maeda K (2000). International recommendations and guidelines for the safe use of diagnostic ultrasound in medicine. Ultrasound Med Biol.

[4] Barzelai S, Sharabani-Yosef O, Holbova R, Castel D, Walden R, Engelberg S (2006). Low-intensity ultrasound induces angiogenesis in rat hind-limb ischemia. Ultrasound Med Biol.

[5] Bertuglia S (2007). Mechanisms by which low-intensity ultrasound improve tolerance to ischemia-reperfusion injury. Ultrasound Med Biol.

[6] Cintas P, Nguyen F, Boneu B, Larrue V (2004). Enhancement of enzymatic fibrinolysis with 2-MHz ultrasound and microtubules. J Thromb Haemost.

[7] Feril LB, Kondo T (2004). Biological effects of low-intensity ultrasound: the mechanism involved, and its implications on therapy on biosafety of ultrasound. J Radiat Res.

[8] Gonçalves WLS, Cirqueira JP, Soares LS, Bissoli NS, Moysés MR (2005). Use of low intensity ultrasonic therapy in the reduction of gynecoid lipodystrofy: a safe therapy or transitory cardiovascular risk? A pre-clinical study. An Bras Dermatol..

[9] Gonçalves WL, Souza FM, Conti CL, Cirqueira JP, Rocha WA, Pires JG (2007). Influence of He-Ne laser therapy on the dynamics of wound healing in mice treated with anti-inflammatory drugs. Braz J Med Biol Res.

[10] Guirro R, Elias D, Serrão F, Bucalon AJ (1996). Dosimetria de aparelhos de ultra-som terapêutico utilizando balança semi-analítica. Rev Bras Fisiot.

[11] Iida K, Luo H, Hagisawa K, Akima T, Shah PK, Naqvi TZ (2006). Noninvasive low-frequency ultrasound energy causes vasodilation in humans. J Am Coll Cardiol.

[12] Khattab AA, Brodersen B, Schuermann-Kuchenbrandt D, Beurich H, Tölg R, Geist V (2007). Extracorporeal cardiac shock wave therapy: first experience in the everyday practice for treatment of chronic refractory angina pectoris. Int J Cardiol.

[13] Kogure A, Yoshida T, Takakura Y, Umekawa T, Hioki C, Yoshioka K (2005). Effect of ultrasonic stimulation on mRNA abundance of uncoupling protein (UCP) 2 and UCP 3 in gastrocnemius muscle of rats. Clin Exp Pharmacol Physiol.

[14] Kuma F, Ueda N, Ito H, Maruyama T, Kaji Y, Fujino T (2006). Effects of ultrasound energy application on cardiac performance in open-chest guinea pigs. Circ J.

[15] Lauer CG, Burge R, Tang DB, Bass BG, Gomez ER, Alving BM (1992). Effect of ultrasound on tissue-type plasminogen activator-induced thrombolysis. Circulation.

[16] Miyamoto T, Neuman Y, Luo H, Jeon DS, Kobal S, Ikeno F (2003). Coronary vasodilation by noninvasive transcutaneous ultrasound: an in vivo canine study. J Am Coll Cardiol.

[17] Miwa H, Kino M, Han LK, Takaoka K, Tsujita T, Furuhata H (2002). Effects of ultrasound application on fat mobilization. Pathophysiology.

[18] Noble JG, Lee V, Griffith-Noble F (2007). Therapeutic ultrasound: the effects upon cutaneous blood flow in humans. Ultrasound Med Biol.

[19] Petrishchev NN, Vlasov TD, Galagudza MM, Makov YN, Minasyan CM (2003). Frequency dependent effects of low-intensity ultrasound on activity of isolated heart. Bull Exp Biol Med.

[20] Rosenthal I, Sostaric JZ, Riesz P (2004). Sonodynamic therapy--a review of the synergistic effects of drugs and ultrasound. Ultrason Sonochem.

[21] Rosim GC, Barbieri CH, Lanças FM, Mazzer N (2005). Diclofenac phonophoresis in human volunteers. Ultrasound Med Biol.

[22] Siegel RJ, Suchkova VN, Miyamoto T, Luo H, Baggs RB, Neuman Y (2004). Ultrasound energy improves myocardial perfusion in the presence of coronary occlusion. J Am Coll Cardiol.

[23] Suchkova VN, Baggs RB, Francis CW (2000). Effect of 40-kHz ultrasound on acute thrombotic ischemia in a rabbit femoral artery thrombosis model: enhancement of thrombolysis and improvement in capillary muscle perfusion. Circulation.

[24] Suchkova VN, Baggs RB, Sahni SK, Francis CW (2002). Ultrasound improves tissue perfusion in ischemic tissue through a nitric oxide dependent mechanism. Thromb Haemost.

[25] ter Haar G (2007). Therapeutic applications of ultrasound. Prog Biophys Mol Biol.

[26] Gonçalves WLS, Cirqueira JP, Abreu GR, Moysés MR (2009). Implications of dermosonic lipoclasis for energy metabolism and body composition of healthy Wistar rats. Rev Bras Fisioter.

[27] Coiado OC (2012). Efeitos do ultrassom de potência sobre o coração: experimentos in vitro e in vivo.

[28] Buiochi EB (2011). Estudo da interação do tecido cardíaco com o ultrassom.

[29] Freitas EA (2010). Efeitos da terapia ultrassônica de baixa intensidade sobre o infarto agudo do miocárdio em ratos.

[30] Rocha WA, Rodrigues KM, Pereira RR, Nogueira BV, Gonçalves WL (2011). Acute effects of therapeutic 1-MHz ultrasound on nasal unblocking of subjects with chronic rhinosinusitis. Braz J Otorhinolaryngol.

[31] Speed CA (2001). Therapeutic ultrasound in soft tissue lesions. Rheumatology (Oxford).

[32] Siegel RJ, Atar S, Fishbein MC, Brasch AV, Peterson TM, Nagai T (2000). Noninvasive, transthoracic, low-frequency ultrasound augments thrombolysis in a canine model of acute myocardial infarction. Circulation.

